# Challenging Nasal Pathologies: An Inverted Nasal Papilloma Case Series Illustrating Diagnostic Challenges and Management Strategies

**DOI:** 10.7759/cureus.66577

**Published:** 2024-08-10

**Authors:** Hasan F Buali, Hesham Alrayyes, Hamad Habib, Sameer Ansari

**Affiliations:** 1 Medicine, King Hamad University Hospital, Busaiteen, BHR; 2 Otolaryngology - Head and Neck Surgery, King Hamad University Hospital, Busaiteen, BHR; 3 General Practice, King Hamad University Hospital, Busaiteen, BHR; 4 Pathology, King Hamad University Hospital, Busaiteen, BHR

**Keywords:** ent surgery, nasal and sinus, nose neoplasms, sinonasal tumor pathology, benign sinonasal tumour

## Abstract

The sinonasal inverted papilloma is a benign tumor located in the sinuses lining the nasal cavity. It is a very rare tumor, representing approximately 4% of all sinonasal tumors. The incidence of sinonasal inverted papilloma is higher in males than females and is most commonly diagnosed in the 5th decade of life.

Four separate cases of sinonasal pathology involving inverted papillomas are presented in this case series. The first clinical case presents a 68-year-old man with persistent nasal symptoms, who was found to have a sinonasal papilloma, which was successfully removed surgically. In the second case, a 61-year-old woman needed multiple procedures for a comprehensive surgical approach due to her inverted papilloma. Despite postoperative complications, the patient showed improvement at later follow-up appointments. In the third case, a 65-year-old man who had an inverted nasal papilloma previously needed surgery to remove and clear the tumor after developing acute sinusitis and rhinosinusitis symptoms. Last but not least, a 57-year-old male presented with nasal blockage and purulent discharge. Polyps were observed during the examination. The initial biopsy indicated the presence of chronic inflammatory polyps. However, during the operation, a first sample biopsy revealed an inflammatory polyp, but due to the appearance of the mass, the surgeon became suspicious and decided to take another biopsy. The second biopsy confirmed the presence of an inverted nasal papilloma. All things considered, these cases demonstrate clinical variability, difficulties in diagnosing, and effective management techniques related to inverted and sinonasal papillomas.

The aim of this case series is to emphasize the importance of proper history taking, physical examination, and use of diagnostic tools to distinctly diagnose inverted nasal papilloma as its symptoms are similar to rhinosinusitis, especially chronic rhinosinusitis.

## Introduction

Sinonasal inverted papilloma, a benign lesion in the nasal cavity and paranasal sinuses, can emerge from the septum or nasal vestibule as fungiform papillomas or from the lateral nasal wall or paranasal sinus mucosal surface as inverted and cylindrical papillomas [1]. Inverted papillomas are rare tumors comprising approximately 4% of all sinonasal tumors. They predominantly occur unilaterally, and exhibit a higher prevalence in males during their fifth decade of life [2]. Uncommon presentations of the inverted papilloma, such as bilateral involvement, were observed in around 5% of cases [3]. Various causes, such as viral infection, smoking, and chronic inflammation, all lead to the formation of nasal inverted papilloma [4]. The most common symptom is unilateral nasal obstruction, and on inspection, unilateral masses with a polypous appearance are commonly seen. When compared to inflammatory polyps, these masses seem opaque and have a coarser texture [5]. Sinonasal inverted papilloma is associated with three key biological characteristics: a proclivity for recurrence, the possibility for local tissue loss, and a proclivity for malignant change in approximately 3-10% of cases [6]. Before definitive resection of sinonasal inverted papilloma, which can often be done in a clinical setting, tissue histopathology should be acquired. Preoperative imaging, such as non-contrast CT scans and magnetic resonance imaging (MRI), is commonly utilized to detect tumor extent and aid in surgical planning, with MRI surpassing CT in distinguishing tumors from secretions and predicting malignant change [7]. Follow-up for sinonasal inverted papilloma is not standardized, but experts suggest a minimum of three to five years. This should include a clinical examination and a regular flexible endoscopy. Biopsies may be performed if there are concerns about recurrence [7]. Although there is a lack of a widely accepted classification system for nasal inverted papillomas, the Krouse classification focuses on the extent of the inverted papillomas and is known for its ease of implementation and reproducibility, and it has been widely used [8]. Surgery is the most effective treatment strategy for nasal inverted papillomas and involves a variety of procedures, including both external and endoscopic approaches. While the external method can cause discomfort, facial scarring, deformities, and oral fistulas, the endoscopic method offers advantages such as no incisions on the face, reduced scaling, facial swelling, preservation of healthy tissue, and a reduced bleeding rate [9]. The most significant risk factor for recurrence in sinonasal inverted papilloma is incomplete surgical resection of the primary lesion site [10].

## Case presentation

Case 1

A 68-year-old male, a known case of diabetes mellitus, hypertension, and hyperlipidemia, presented to the otolaryngology clinic with complaints of nasal symptoms for three years. His main symptoms were nasal blockage causing sleep disturbance, bloody discharge from the nose, sometimes difficulty breathing, especially when lying down, and occasional snoring and mouth breathing. He denied any hyposmia, headache, or facial pain; there was no observed apnea, and there were no other complaints. The patient went to a private hospital, and he gave a history of turbinoplasty coblation five months ago, but without improvement in his symptoms. The patient was a non-smoker and had no allergies or family history of similar conditions.

A physical examination was done, and there were no visible or palpable neck lymph nodes or masses. The thyroid gland was not enlarged, there were no nodules on the gland, and the gland was not tender to the touch. The throat showed normal-sized tonsils with no signs of inflammation. The ear was a little bit waxy. There was a mild right-sided nasal deviation and mildly enlarged turbinates. A mass was seen on a nasal endoscopy descending from the left ostiomeatal complex and blocking the left choana.

The patient was advised to undergo a biopsy, and a CT scan was requested. Nasal decongestants and antihistamines were prescribed while waiting for the CT scan and biopsy.

After three weeks, the patient presented to the clinic again for follow-up. The CT scan result showed a soft tissue density lesion in relation to the left nasal cavity posterior and lower aspect, which could be suggestive of neoplastic mass or polyposis. Slightly deviated nasal septum (DNS) to the right was noted. He had mildly hypertrophied both inferior turbinates, with bilateral concha bullosa. Mucosal occlusion of the right ostiomeatal complex and partial occlusion of the left ostiomeatal complex were noted, and Haller's cells were seen on the left.

**Figure 1:**
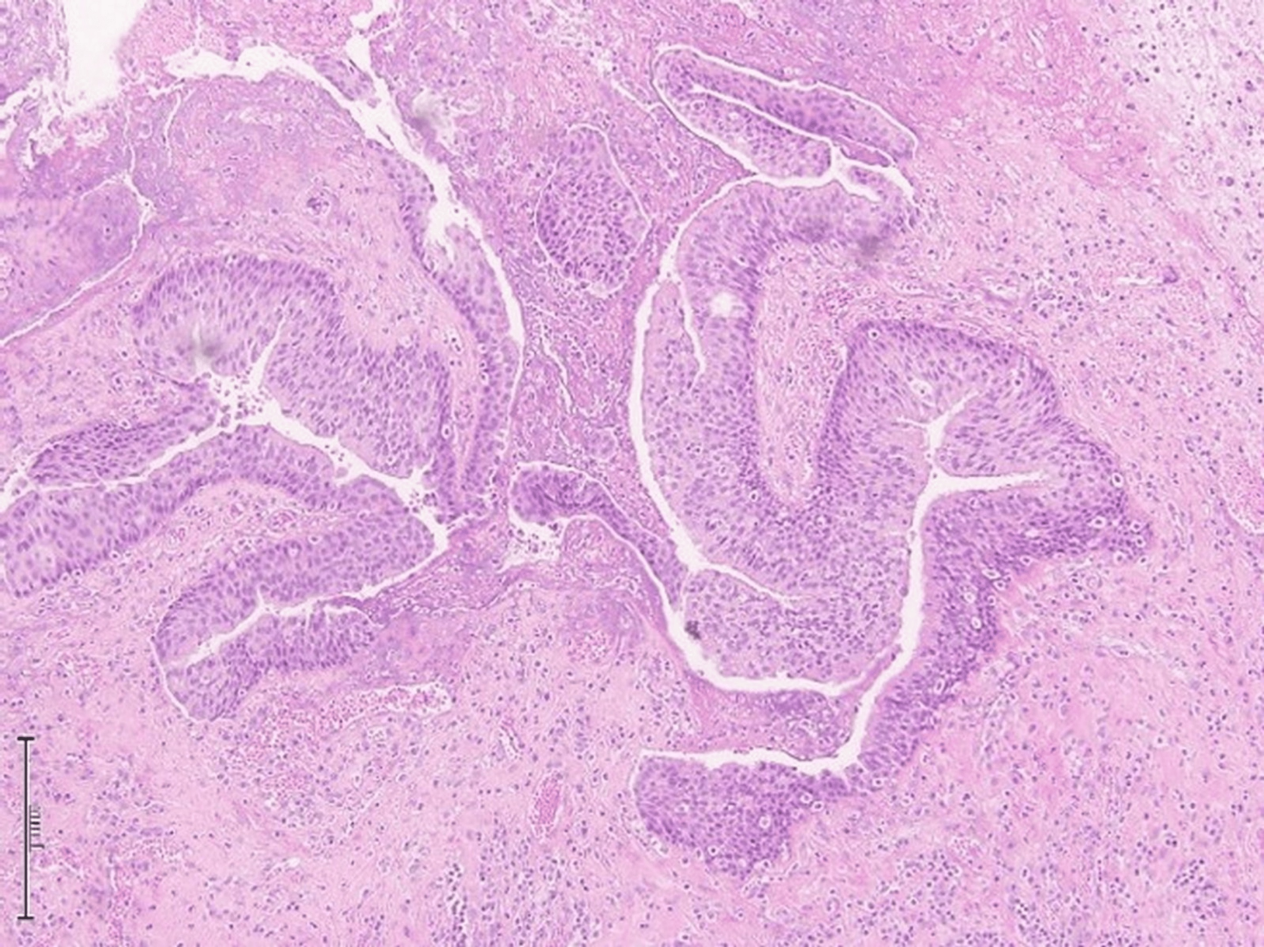
Hematoxylin and eosin (H&E) stain, 20x magnification. The microphotograph revealed prominent downward endophytic growth of round to elongated interconnected epithelial nests with smooth outer contours.

**Figure 2:**
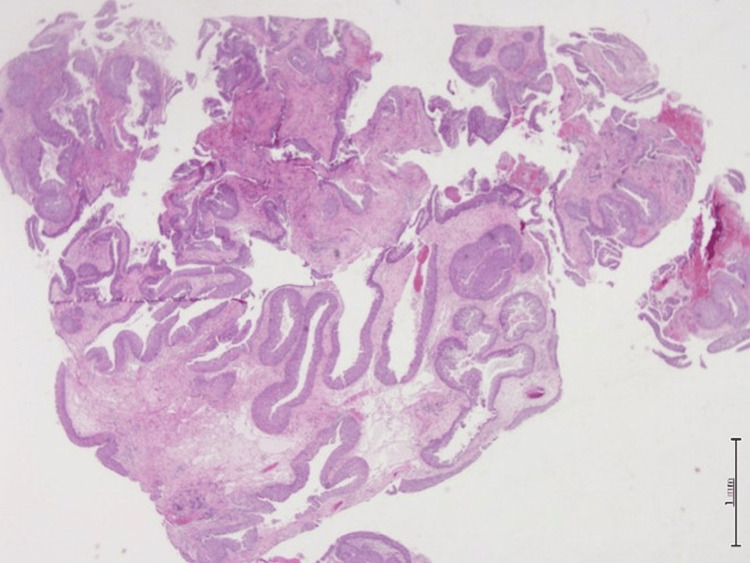
Hematoxylin and eosin (H&E) stain, 2x magnification. The epithelium is hyperplastic and of squamous type with overall maintained polarity with transmigration of neutrophils seen with stroma edema or chronic inflammation.

The histopathology reports showed a left posteroinferior nasal cavity mass, most likely sinonasal papilloma, exophytic type (Figures 1, 2). The patient was booked for functional endoscopic sinus surgery (FESS). The procedure was done under general anesthesia; the left concha was released under endoscopic guidance; the left wide antrostomy was done; dissection through crista ethmoidalis was done; and the cauterization of the sphenopalatine artery was done with suction diathermy. Excision of the mass was done with the removal of the attaching mucosa and debriding of the inferior part of the middle turbinate, and the mass was sent for biopsy. The post-excisional biopsy showed an inverted nasal papilloma with no dysplasia or malignancy.

Currently, the patient is recovering from the surgery, and the prognosis is good since the biopsy showed no dysplasia or malignancy. Following up after six months is indicated as per protocol.

Case 2

A 61-year-old female patient with a known history of diabetes mellitus was referred from the radiation oncology department as a case of inverted papilloma with multiple foci with a history of being operated on around five to six times. During the examination, bilateral polypoidal masses were observed. A CT scan of the sinuses revealed polypoidal mucosal thickening in the right maxillary sinus, extending into the right side of the nasal cavity. Mild involvement of the remaining ethmoidal air cells, frontal sinus, sphenoid sinus, and bilateral Onodi cells was also noted.

The patient underwent surgery under aseptic conditions. The nasal cavity was packed with adrenaline ribbon gauze, findings on the right side were suggestive of benign sinus disease, and a right-sided antrostomy was performed to remove a retention cyst and polyps. Additionally, a large septal perforation with posterior septal polyps was identified. The left side lesion was highly suspicious of recurrent inverted papilloma as confirmed by biopsy at the OPD, extensively involving maxillary antrum at multiple seeding sites. The left-sided papilloma was removed using a microdebrider, accompanied by bone drilling. To improve access to the tumor, a combined approach of the Caldwell-Luc procedure via sublabial incision involving extensive bone drilling and endoscopic procedure was performed. Further interventions included anterior and posterior ethmoidectomy, sphenoidotomy, left-sided dacryocystorhinostomy (DCR) with stent application, closure of the oral incision using Vicryl sutures, and bilateral application of NasoPore.

**Figure 3:**
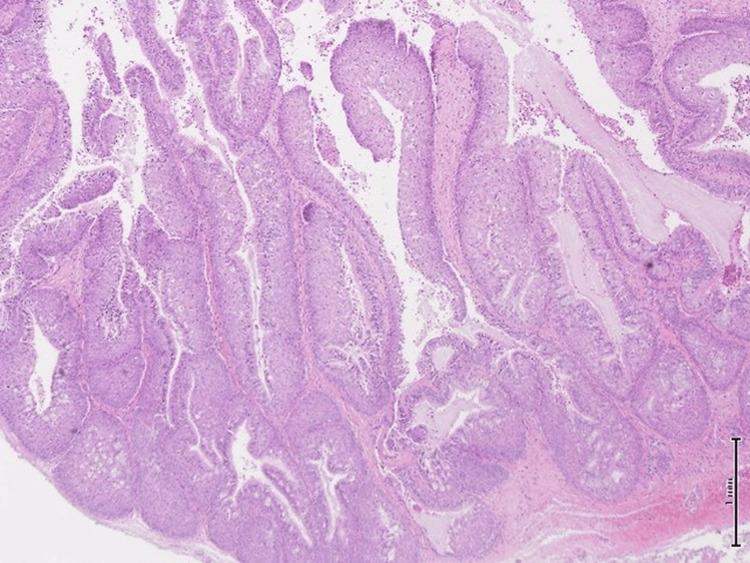
Hematoxylin and eosin (H&E) stain, 10x magnification. The microphotograph revealed prominent downward endophytic growth of round to elongated interconnected epithelial nests with smooth outer contours.

**Figure 4:**
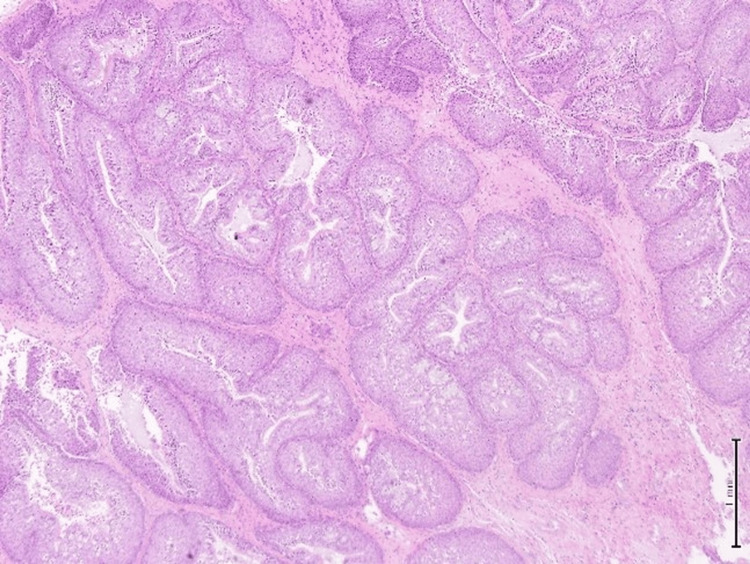
Hematoxylin and eosin (H&E) stain, 10x magnification. The epithelium is hyperplastic and of squamous type with overall maintained polarity with transmigration of neutrophils seen.

No intraoperative or postoperative complications were encountered, and the patient was discharged home. The postoperative biopsy results revealed the following findings: chronic inflammation in the nasal biopsy and right posterior septum; an inflammatory nasal polyp in the right maxillary antrum; inverted papillomas in the left ethmoid, left maxillary antrum, left anterior maxillary, left antrum, and lateral nasal wall; fibrofatty tissue in the subcutaneous tissue; mild chronic inflammation in the floor of the nose; and inverted papilloma involving the lamina papyracea (Figures 3, 4).

Ten days postoperatively, the patient presented with numbness and pain over the maxillary teeth. There was no numbness of the cheek. Two weeks later, she complained of water regurgitation from the nose without any issues with solid oral intake. Examination revealed satisfactory healing of the left-sided incision. After re-evaluation two weeks later, water leakage from the wound persisted, which is highly suggestive of an oro-antral fistula because of the sublabial incision with the Caldwell-Luc procedure. Restitching was performed, and saline douches were advised. One week later, she reported improvement and no leakage. Nasal endoscopy showed clear sinus openings. During the follow-up visit one week later, she continued to do well. Nasal cleaning was performed, crusts were suctioned, and endoscopic examination revealed clear sinus openings. Three weeks later, she complained again of water leakage from the left oral wound. The patient was admitted, and closure of the left oro-antral fistula was carried out. During the follow-up visit one week later, she had no active complaints, and the wound site appeared clean with intact sutures. At the three-month follow-up, the patient remained asymptomatic. Nasal endoscopy revealed a large septal perforation and clear sinuses without signs of recurrence. An MRI was performed, showing significant regression of previously observed diffuse polypoidal sino-nasal masses with features of postoperative changes.

Case 3

A 65-year-old male with a known case of inverted nasal papilloma underwent two FESS procedures under a different surgeon. He presented to the clinic with complaints of left facial pain and fullness in the temple, forehead, and maxillary area, along with throat dryness and nighttime discomfort. On examination, a DNS to the left and congested mucosa in the throat was observed. A provisional diagnosis of rhinosinusitis with DNS was made.

Three months later, the patient returned with a complaint of crusts coming from the nose. A flexible nasoendoscopy (FNE) examination showed no active pus or identifiable mass. The patient was prescribed nasal antihistamines and decongestants. One month later, he complained of severe left-sided nasal blockage, headache, and foul-smelling discharge. A CT scan of the sinuses was ordered prior to the third operation, and the patient was prescribed oral antibiotics for one week along with nasal douches. The CT scan revealed the presence of an air-fluid level in the left maxillary sinus, indicating acute sinusitis. Additionally, there was an increase in mucosal thickening in the bilateral frontal and ethmoid cells, nasal cavity walls, and septum, suggesting increased inflammatory changes. Hypertrophy of the bilateral inferior turbinates and a narrowed right maxillary ostium with mucosal thickening were also noted.

**Figure 5:**
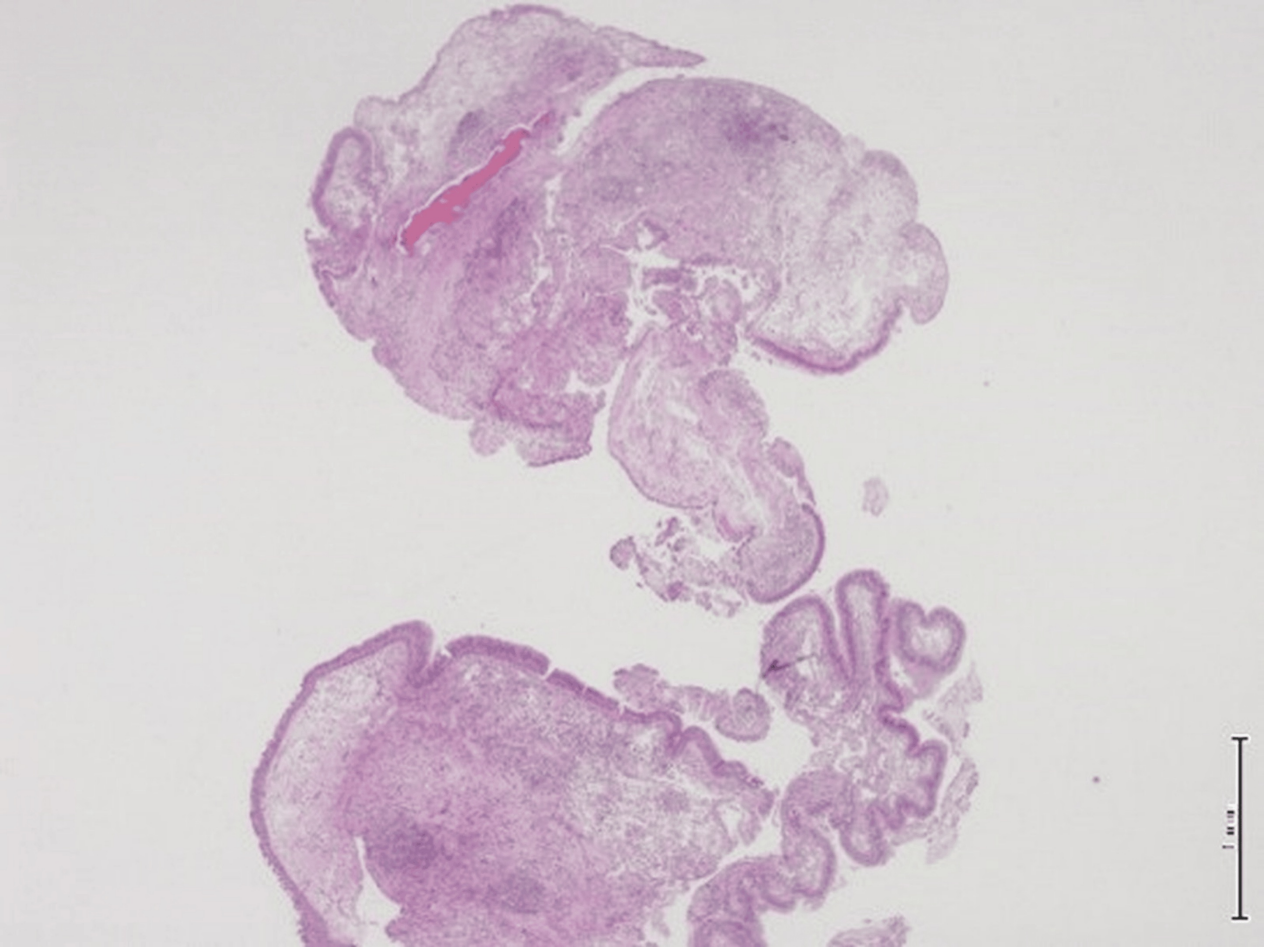
Hematoxylin and eosin (H&E) stain, 10x magnification. The microphotograph revealed prominent downward endophytic growth of round to elongated interconnected epithelial nests with smooth outer contours.

**Figure 6:**
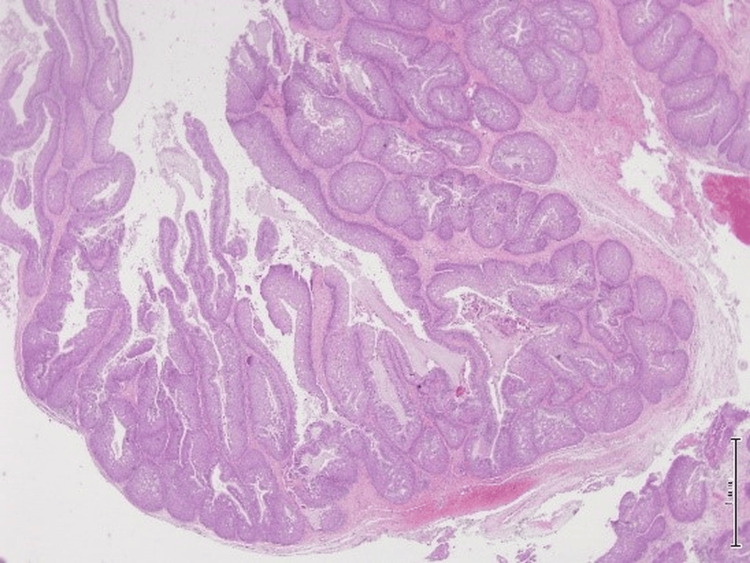
Hematoxylin and eosin (H&E) stain, 4x magnification. The microphotograph showed a polyp lined by benign pseudostratified ciliated columnar epithelium with edematous stroma with mixed inflammation.

The patient was scheduled for surgery. Under aseptic measures, the nasal cavity was packed with xylometazoline. The left middle turbinate was excised and sent for histopathological examination. An intraoperative frozen section obtained from the nasal cavity confirmed the diagnosis of inverted nasal papilloma (Figures 5, 6). The left maxillary antrum was found to be patent, and the facial recess was opened. A Draf IIB procedure was performed to gain access and view the tumor. The tumor was reached, which was found to be arising from the anterior and lateral frontal walls. The tumor was carefully excised using debridement and coblation. A residual tumor in the ethmoid region was also effectively cleared. A silastic sheet was applied to the frontal sinus, and hemostasis was ensured using suction cautery.

The patient returned for follow-up visits, which were uneventful, and the patient was doing well. Nasal cleaning was performed, the stent was removed, and crusts were cleared. No nasal obstruction, snoring, or sleep apnea was reported. Scopes were performed, and clear sinuses were observed with no evidence of recurrence.

Case 4

**Figure 7:**
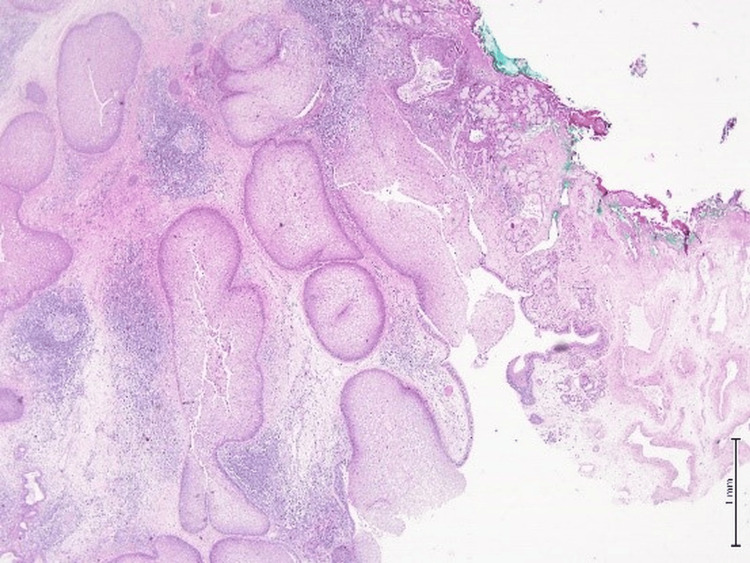
Hematoxylin and eosin (H&E) stain, 4x magnification. The epithelium is hyperplastic and of squamous type with overall maintained polarity with transmigration of neutrophils seen. Stroma edema or chronic inflammation and surrounding benign seromucous glands are seen.

**Figure 8:**
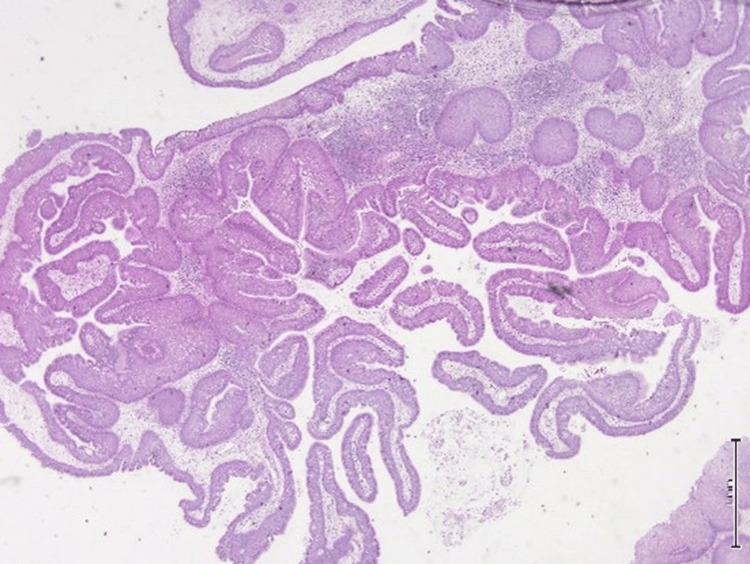
Hematoxylin and eosin (H&E) stain, 4x magnification. The microphotograph revealed prominent downward endophytic growth of round to elongated interconnected epithelial nests with smooth outer contours.

A 57-year-old male with no known medical history presented to the clinic complaining of nasal blockage. Upon examination, the patient exhibited purulent discharge on the right side and bilateral grade 3 polyps. The treatment plan included prescribing augmentin, nasal wash, and nasal steroids. A biopsy was performed in the clinic, revealing a chronic inflammatory "allergic" polyp. Additionally, a CT sinus scan showed heterogeneous soft tissue opacification in the right maxillary and right ethmoid sinuses, raising suspicion of a right inverted papilloma or antrochoanal polyp. The patient was scheduled for FESS under general anesthesia. During the procedure, bilateral Otrivin packs were inserted, and a right-sided medial maxillectomy was performed. The mass was found to extend from the maxillary antrum, attaching to the posterior part of the turbinate and posterior septum bilaterally, with the involvement of the frontal sinus recess. Diathermy was used to remove the mass and achieve hemostasis. The resected mass was sent for histopathology, initially revealing an inflammatory polyp on the frozen section (Figure 7). However, the mass extended down to the nasopharynx and had attachments in the posterior part of the vomer, which made suspicion of inverted nasal papilloma higher. Another frozen section was obtained, which confirmed the presence of sinonasal papilloma, specifically the inverted type (Figure 8). Because of the intraoperative findings and frozen section result of inverted papilloma, the surgeon performed a medial maxillectomy involving bone drilling. The patient remained stable throughout the procedure and was handed over to the anesthesia team. Postoperative biopsy results showed an ethmoid sinus mass of inflammatory nasal polyp, the right maxillary sinus mass showed sinonasal papilloma, inverted type, and the right frontal recess mass showed sinonasal papilloma, inverted type. Currently, the patient is recovering well and following the recommended postoperative protocol.

## Discussion

In this case series, we present four distinct cases of nasal papillomas, each with its unique clinical features and outcomes. Each case also has its unique set of challenges and difficulties in the diagnosis and treatment of the papilloma.

The first case was about a 68-year-old male patient with a history of diabetes mellitus, hypertension, and hyperlipidemia. The patient had nasal congestion leading to sleep disturbances, bloody nasal discharge, difficulty in breathing when lying down, and occasional snoring and breathing through the mouth. On physical examination, a slight nose deviation to the right was noted, as well as a mass obstructing the left choana during nasal endoscopy. FESS was performed to remove the tumor and attached mucosa. The removal of the nasal papilloma was more difficult than usual because the patient had previously undergone unnecessary turbinoplasty. The turbinoplasty had changed the anatomy of the nasal turbinates, making it harder to reach and remove the papilloma. However, the surgeon was able to carefully plan and execute the surgery, and the papilloma was successfully removed along with the surrounding tissue.

The second and third cases emphasize the importance of complete excision of inverted nasal papillomas to prevent recurrence. In the second case, a 61-year-old female presented with an inverted papilloma involving multiple sinus regions. Despite successful surgical intervention, the patient experienced symptoms of water regurgitation from the nose without any issues with solid oral intake. This suggests that there was communication between the nasal cavity (being maxillary antrum) and the oral cavity because of the sublabial incision performed during surgery, leading to the passage of liquids through an oro-antral fistula. The water leakage persisted despite initial attempts at conservative management. The surgeon opted to perform stitching under sedation or general anesthesia to close the left oro-antral fistula. Subsequent follow-up visits showed positive outcomes, indicating the significance of thorough removal. Similarly, in the third case, a 65-year-old male with a previous diagnosis of inverted nasal papilloma developed acute sinusitis due to a residual tumor. Surgical intervention was necessary to remove the remaining tumor and achieve symptomatic relief. It is important to note that incomplete surgical excision is a significant risk factor for the recurrence of sinus-inverted papilloma [10]. Therefore, regular follow-up examinations, including flexible endoscopy, are recommended to monitor and detect any signs of recurrence.

The fourth case involved a 57-year-old male who initially presented with nasal blockage and purulent discharge. Bilateral grade 3 polyps were observed during the examination. The initial biopsy indicated the presence of chronic inflammatory "allergic" polyps, and the patient was scheduled for surgery. However, during the operation, a first sample biopsy revealed an inflammatory polyp. Due to the complexity and appearance of the mass, the surgeon became suspicious and decided to take another biopsy. This second biopsy confirmed the presence of an inverted nasal papilloma. This finding emphasizes the importance of a thorough evaluation and considering differential diagnoses in complex cases. It highlights the need to remain vigilant during surgical procedures and to investigate further when the clinical picture does not align with the initial findings.

This case series highlights the importance of taking a complete history, performing a thorough examination, and using appropriate diagnostic tools, such as nasal endoscopy, for accurate diagnosis and quick removal of the nasal papilloma. Early detection and appropriate surgical intervention are important to manage and prevent potential complications associated with these tumors.

## Conclusions

A rare benign tumor of the paranasal sinuses and nasal cavity is known as a sinonasal inverted papilloma. It carries a risk of malignant transformation, local tissue destruction, and a propensity for recurrence. The most effective course of action is surgery, and endoscopic surgery is the preferred method whenever practical. This case series highlights the importance of taking a complete history, performing a thorough examination, and using appropriate diagnostic tools, such as nasal endoscopy. These measures can help identify specific findings that may indicate the presence of nasal papillomas, leading to a timely diagnosis and treatment.
